# Morpho-Molecular and Ultrastructural Characterization of *Discocriconemella Parasinensis* n. Sp. from Zhejiang Province, China

**DOI:** 10.2478/jofnem-2022-0011

**Published:** 2022-05-18

**Authors:** Junxia Li, Maria Munawar, Pablo Castillo, Jingwu Zheng

**Affiliations:** 1 Laboratory of Plant Nematology, Institute of Biotechnology, Department of Plant Protection, College of Agriculture and Biotechnology, Zhejiang University, Hangzhou, Zhejiang 310058, P.R. China; 2 Department of Biological Sciences, University of Lethbridge, 4401 University Drive West, Lethbridge, Alberta T1K 3M4, Canada; 3 Department of Crop Protection, Institute for Sustainable Agriculture (IAS), Spanish National Research Council (CSIC), Campus de Excelencia Internacional Agrolimentario, ceiA3, Avenida Menendez Pidal s/n, 14004 Cordoba, Spain; 4 Ministry of Agriculture Key Lab of Molecular Biology of Crop Pathogens and Insects, Department of Plant protection, Hangzhou, Zhejiang 310058, P.R. China

**Keywords:** *Camellia sinensis*, DNA sequencing, morphology, morphometrics, nematode, new record, phylogeny, species

## Abstract

During a recent inventory survey of the nematofauna of tea plantation at Zhejiang Province, China, *Discocriconemella parasinensis* n. sp. was detected in the rhizosphere of *Camellia sinensis*. The new species can be characterized by having the uninterrupted rounded labial disc, *en face* view showing rectangular-rounded labial plate without submedian lobes, R = 82.6 (80–86), Rex = 22 (21–23), stylet length of 68.3 (59–76) μm, excretory pore located 1–2 annuli posterior to the esophageal bulb, vulva open, postvulval body elongated conoid, and tail conoid with bilobed terminus. Morphologically, the species shares the same lip-type with *D. discolabia*, *D. mauritiensis*, *D. mineira*, *D. perseae*, and *D. sinensis*. Phylogenetic relationships of the new species based on D2–D3 expansion segments of 28S, ITS, and 18S rRNA genes revealed that *D. parasinensis* n. sp. formed a separated clade from other criconematid species, thereby supporting its status as a new species of the genus. The new species showed close phylogenetic relationships with *Criconemoides geraerti*.

Zhejiang, located in southeast coast of China, is a well-renowned tea producing province and pivotal to the Chinese tea industry in terms of the highest acreage and economic returns (FAO and CAAS, 2021). Over the last 10 years, tea cultivation has been expanded to meet the increased tea consumption and to benefit small-scale growers (Liang et al., 2021). To maintain high standards of tea cultivation, planted fields are regularly surveyed to examine the presence of pest species. The latter involves the collection of soil samples and determining the associated plant-parasitic nematodes. Recent reports have described the incidence of ring nematodes from tea cultivated areas of Zhejiang (Maria et al., 2018a, 2019). Therefore, surveys have been carried out to study the nematofauna of tea plantations in Zhejiang. During our recent survey, we isolated a population of *Discocriconemella* sp. that exhibited uninterrupted labial disc, robust stylet, and conoid elongate posterior body.

*Discocriconemella* (De Grisse and Loof, 1965) includes 29 species, and so far only three species [*D. hengsungica* (Choi and Geraert, 1975); *D. limitanea* (Luc, 1959; De Grisse and Loof, 1965); and *D. sinensis* (Maria et al., 2019)] have been reported from China (Geraert, 2010; Maria et al., 2018a, 2019).

To determine the precise identity of recently isolated specimens, a detailed morphological study on lip region, vulva structure, and tail lobes was conducted using scanning electron microscopy (SEM) and light microscopy. The qualitative and quantitative characteristics of this population were compared with related species, and we found that this species possesses unique characters that support its status as a new species.

Hence, it is described herein as *D. parasinensis* n. sp. with the following objectives: (i) to provide an integrative morphological and molecular characterization of *D. parasinensis* n. sp.; (ii) to elucidate important morphological details through SEM observations; and (iii) to study the phylogenetic relationships of this newly discovered *Discocriconemella* sp. with other criconematid species using 18S, D2–D3 of 28S, and ITS rRNA gene sequences.

## Materials and methods

### Nematode population sampling, extraction, and morphological identification

Nematodes were extracted from soil samples using the Cobb sieving and flotation-centrifugation method (Jenkins, 1964). For morphometric studies, the nematodes were killed and fixed with hot formalin and processed to glycerin (Seinhorst, 1959) as modified by De Grisse (1969). The measurements and light micrographs of nematodes were made with a Nikon Eclipse Ni-U 931845 compound microscope. The drawings were made using a drawing tube attached to the microscope and were redrawn using Corel DRAW software version 16 (Corel). For the SEM examination, 40–50 handpicked nematodes were fixed in a mixture of 2.5% paraformaldehyde and 2.5% glutaraldehyde, washed three times in 0.1M cacodylate buffer, postfixed in 1% osmium tetroxide, dehydrated in a series of ethanol solutions, and critical point-dried with CO_2_. After mounting on stubs, the samples were coated with gold at 6-nm to 10-nm thickness and the micro-graphs were made at 3–5 kV operating system of Hitachi SU8010 (Maria et al., 2018a).

### DNA extraction, PCR, and sequencing

DNA samples were prepared according to Zheng et al. (2003). Four sets of primers (synthesized by Healthy Creatures, Hangzhou, China) were used in the PCR analyses to amplify the near full-length 18S, D2–D3 expansion segments of 28S, and ITS region of rRNA. Partial 18S region was amplified with two sets of primers. The first set was 18s39F (5′-AAAGATTAAGCCATGCATG-3′) and 18s977R (5′-TTTACGGTTAGAACTAGGGCGG-3′), and the second set was 18s900F (5′-AAGACGGACTACAGCGAAAG-3′) and 18s1713R (5′ TCACCTACAGCTACCTTGTTACG-3′) (Olson et al., 2017). Primers for amplification of ITS were F195 (5′-TCCTCCGCTAAATGATATG-3′) and V5367 (5′-TTGATTACGTCCCTGCCCTTT-3′) (Vrain et al., 1992). Primers for amplification of D2–D3 28S were the forward D2A (5′-ACAAGTACCGTGAGGGAAAGTTG-3′) and the reverse D3B (5′-TCGGAAGGAACCAGCTACTA-3′) (De Ley et al., 1999). PCR conditions were maintained as described by Ye et al. (2007) and Powers et al. (2010). PCR products were evaluated on 1% agarose gels and stained with Gelred (Tsingke Biotechnology, TSJ003). PCR products of sufficiently high quality were sent for sequencing to Healthy Creatures. The newly obtained sequences were submitted to the GenBank database under accession numbers indicated on the phylogenetic trees.

### Phylogenetic analyses

Newly obtained sequences of 18S, D2–D3 expansion segments of 28S and ITS gene sequences, and available sequences of other nematodes obtained from GenBank were used for phylogenetic reconstructions of criconematid species. The selection of outgroup taxa for each dataset was based on previously published studies (Maria et al., 2018a, 2019). Multiple sequence alignments of the different genes were completed using the FFT-NS-2 algorithm of MAFFT v7.450 (Katoh et al., 2019). The BioEdit program v7.2.5 (Hall, 1999) was used for sequence alignment visualization and edited using Gblocks v0.91b (Castresana, 2000) using options for a less stringent selection (minimum number of sequences for a conserved or a flanking position: 50% of the number of sequences + 1; maximum number of contiguous non-conserved positions: 8; minimum length of a block: 5; allowed gap positions: with half). Phylogenetic analyses of the sequence datasets were based on Bayesian inference (BI) using MrBayes v3.1.2 (Ronquist and Huelsenbeck, 2003). The best-fit model of DNA evolution was achieved using JModelTest v2.1.7 (Darriba et al., 2012) with the Akaike information criterion (AIC). The best-fit model, the base frequency, the proportion of invariable sites, and the gamma distribution shape parameters and substitution rates in the AIC were then used in MrBayes for the phylogenetic analyses. The transition model with invariable sites and a gamma-shaped distribution of invariable sites (TIM3+I +G, TIM1+I+G) for the D2–D3 segments of the 28S rRNA and 18S, respectively, and the general time-reversible model with invariable sites and a gamma-shaped distribution (GTR+I+G) for the ITS rRNA gene and were run with four chains for 4 × 10^6^ generations, respectively. A combined analysis of the two ribosomal genes was not undertaken because several sequences are not available for all species. The sampling for Markov chains was carried out at intervals of 100 generations. For each analysis, two runs were conducted. After discarding burn-in samples of 30% and evaluating convergence, the remaining samples were retained for more in-depth analyses. The topologies were used to generate a 50% majority-rule consensus tree. On each appropriate clade, posterior probabilities (PP) were given. FigTree software v1.4.3 (Rambaut, 2016) was used for visualization of trees from all analyses.

## Results and Description

### Systematics

**Figure 1:**
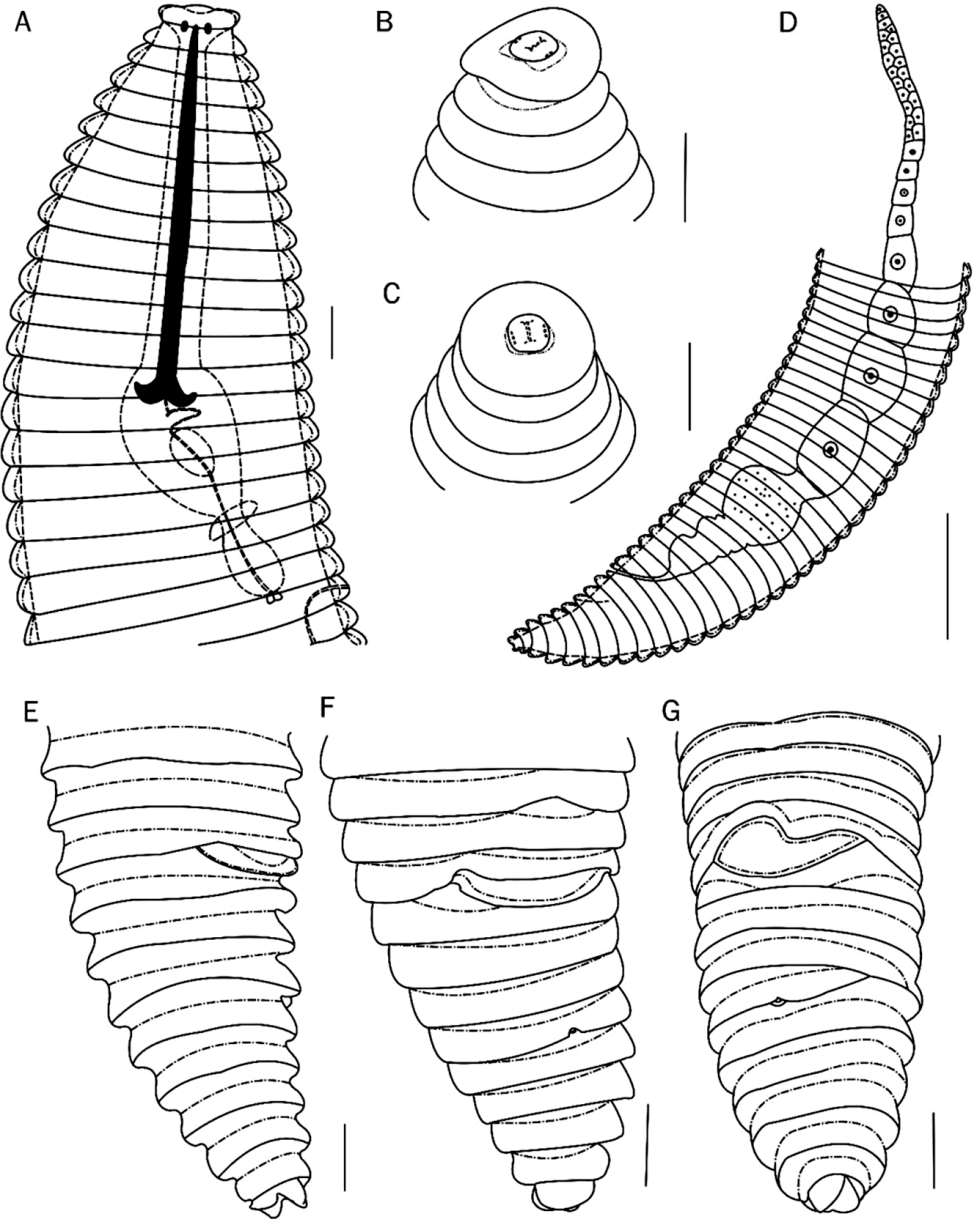
Line drawings of *D. parasinensis* n. sp. Female. (A) Esophageal region; (B, C) *En face* view of lip region; (D) Gonad; (E–G) Posterior region showing position of vulva and anus (Scale bars: A–C, E–G = 10 μm, D = 50 μm).

**Figure 2:**
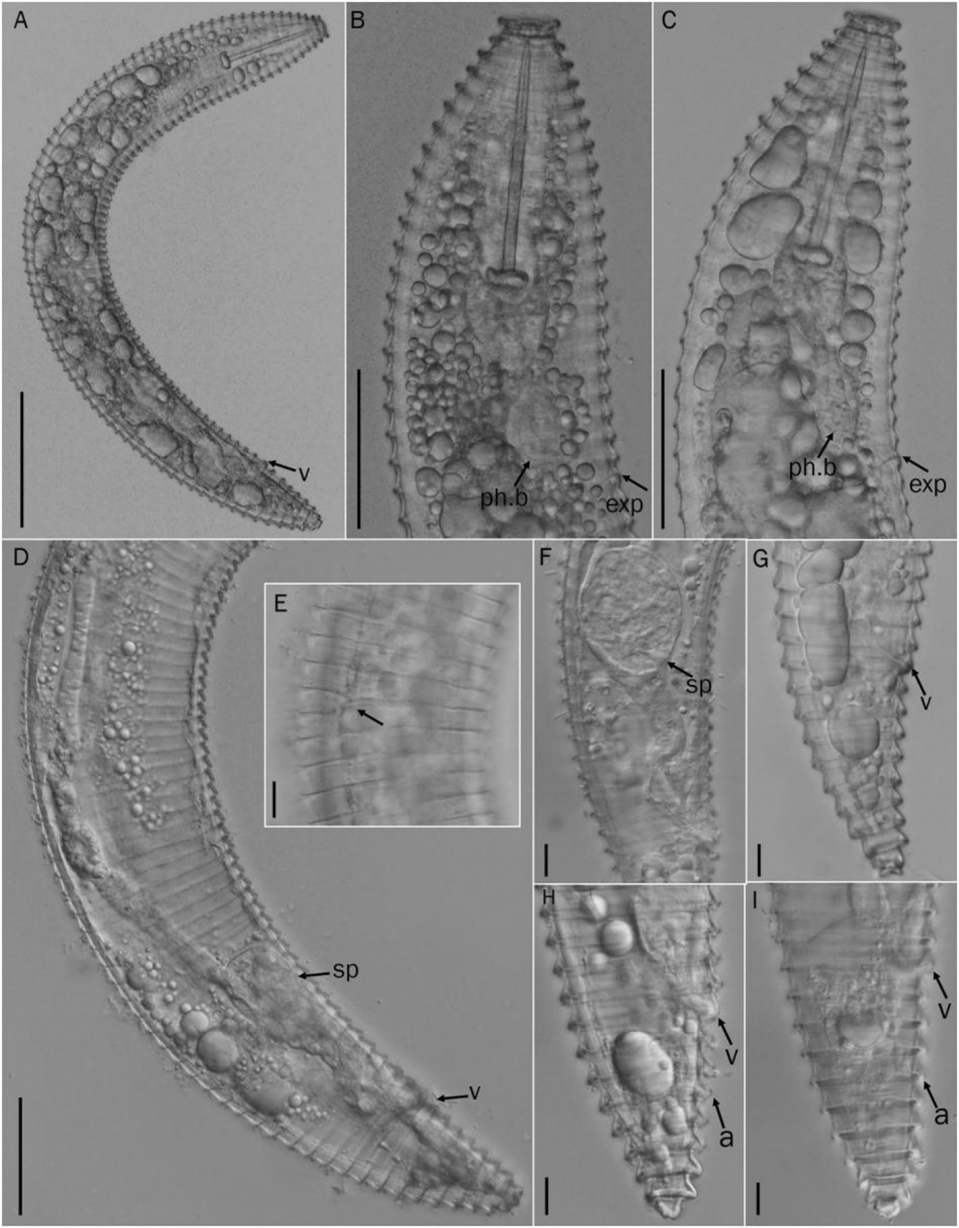
Light photomicrographs of *D. parasinensis* n. sp. Female. (A) Entire body, arrowhead indicating position of vulva (v = vulva); (B, C) Esophageal region arrowheads indicating position of base of esophageal bulb (ph.b) and excretory pore (exp); (D) Posterior region showing entire gonad arrowheads indicating position of vulva (v) and spermatheca (sp); (E) Mid-body (arrowhead pointing anastomoses); (F) Vulval region arrowhead showing spermatheca (sp); (G–I) Tail region, arrowheads showing position of vulva (v) and anus (a) (Scale bars: A = 100 μm, B–D = 50 μm, E–I = 10 μm).

**Figure 3:**
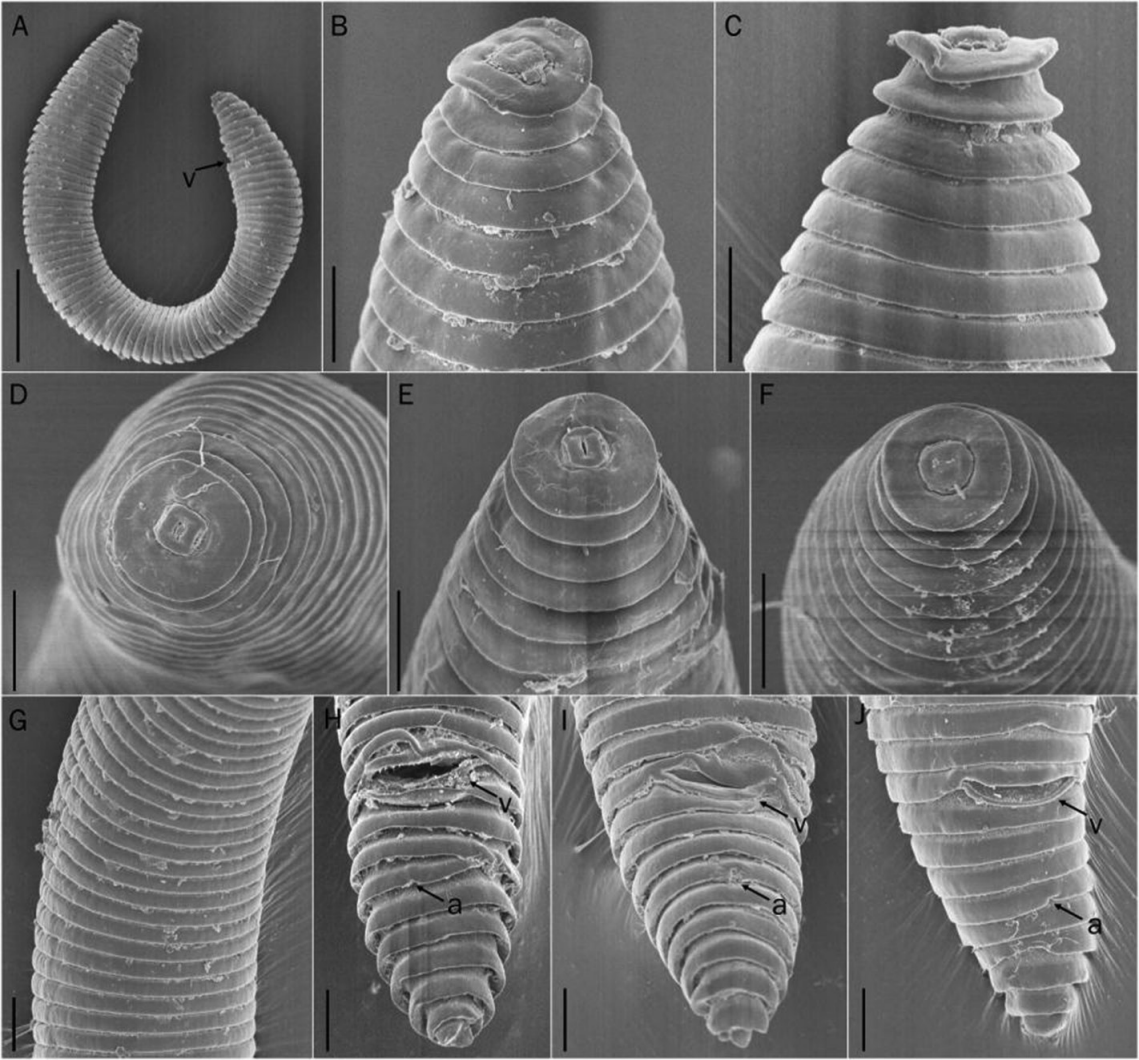
Scanning electron micrographs of *D. parasinensis* n. sp. Female. (A) Entire body, arrowhead indicating position of vulva (v = vulva); (B–F) Lip regions; (G) Cuticular annuli at mid-body; (H–J) Tail region, arrowheads showing position of vulva (v) and anus (a) (Scale bars: A = 50 μm; B–J = 10 μm).

*Discocriconemella parasinensis* n. sp. (Figs. 1–3 and Table 1).

**Table 1:** Morphometric data of female of *D. parasinensis* n. sp.

**Character**	**Holotype**	**Paratype**
n	–	15
L	477.0	486.1 ± 37.6 (382–556)
a	7.8	7.7 ± 0.5 (6.7–9.0)
b	4.0	4.3 ± 0.3 (3.6–4.6)
c	15.5	16.4 ± 2.0 (13.8–20.6)
c′	1.1	1.0 ± 0.1 (0.9–1.3)
V	89.6	89.6 ± 0.8 (88.0–91.0)
VL/VB	1.3	1.2 ± 0.1 (1.1–1.5)
R	82	82.6 ± 1.9 (80–86)
Rex	22	22.0 ± 0.7 (21.0–23.0)
RV	10	10.1 ± 0.4 (9–11)
RVan	3	2.9 ± 0.2 (2–3)
Ran	6	6.1 ± 0.3 (6–7)
Lip height	9.0	7.8 ± 0.6 (7.0–9.0)
Lip diam.	19.0	20.0 ± 1.0 (18.0–22.0)
Stylet length	68.0	68.3 ± 4.9 (59.0–76.0)
Stylet (% L)	14.3	14.1 ± 1.3 (12.3–16.8)
Pharynx	119.0	114.0 ± 5.8 (103.0–123.0)
Max. Body diam.	61.0	63.2 ± 6.3 (51.0–72.0)
Vulval body diam.	38.0	40.9 ± 3.6 (33.0–47.0)
Vulva to tail terminus	49.0	50.4 ± 4.4 (41.0–56.0)
Anal body diam.	28.0	30.0 ± 3.5 (24.0–35.0)
Tail length	31.0	30.0 ± 3.9 (23.0–36.0)

All measurements are in μm and in the form of mean ± SD (range).

### Description

#### Female

Body habitus C-shape after heat relaxation. Cuticle finely annulated, annuli retrorse with posterior margins smooth. A few anastomoses present at mid or posterior body. The first cephalic annulus enlarged disc like. *En face* view showed a circular uninterrupted disc without submedian lobes. Lip pattern matching that of group 1 proposed for *Discocriconemella* species by Vovlas (1992). Labial plate rounded to rectangular in shape slightly raised, with slit-like oral apertures. Stylet rigid and robust, with anchor shaped stylet knobs. Dorsal esophageal gland opening posterior to stylet base esophageal components and general morphology is criconematid type. Esophageal lumen looped in median esophageal bulb, which has medium-sized refractive valvular apparatus. Isthmus narrow, short, encircled by nerve ring. Basal esophageal bulb elongated. Excretory pore conspicuous, situated 1–2 annuli posterior to esophageal bulb. Vulva open (SEM observation), without vulval flaps, the anterior lip slightly overhangs and covers the preceding annuli. Vagina straight, extending for less than half of body diam. Spermatheca rounded to oval, filled with few sperm cells. Gonad single, prodelphic. Oocytes arranged in single file except for a short region of multiplication near anterior end. Anus small, indistinct located 2–3 annuli posterior to vulva. Postvulval body elongated conoid. Tail short, conoid, with a bilobed terminus.

#### Male and juvenile

Not studied.

### Type Host and Locality

The new species was detected in association with *Camellia sinensis* (L.) Kuntze, 1887 from Zijingang Campus of Zhejiang University, Hangzhou, Zhejiang Province, P.R. China. The geographical position of the sampling site is 120°4′48″E, 30°18′36″N.

### Type material

Holotype female and 10 female paratypes (slide numbers ZJU-32-01 to ZJU-32-10) deposited in the nematode collection of Zhejiang University, Hangzhou, P.R. China. Five female paratypes were deposited at the USDA nematode collection, Beltsville, MA, USA. The Zoobank code for the new species is as follows: LSID: zoobank.org:act:49F1E049-BA02-46B1-82EF-304F1F40390B.

### Etymology

The specific epithet *D. parasinensis* formed from the Latin word para = beside or near, and the species epithet *sinensis*, thereby reflecting its close morphological similarity to *D. sinensis*.

### Diagnosis and relationships

*Discocriconemella parasinensis* n. sp. can be characterized by having the uninterrupted rounded labial disc, *en face* view showing rectangular-rounded labial plate without submedian lobes. Total number of body annuli (R = 82.6 [80–86]), number of annuli from anterior end to excretory pore (Rex = 22 [21–23]), stylet length of 68.3 (59–76) μm, excretory pore located 1–2 annuli posterior to the esophageal bulb, vulva open, postvulval body elongated conoid, tail conoid with bilobed terminus.

Based on the lip pattern scheme proposed by Vovlas (1992), this new species belongs to group 1 in having a rounded uninterrupted labial disc. It shares the same lip type with *D. discolabia* (Diab and Jenkins, 1966; De Grisse, 1967), *D. mauritiensis* (Williams, 1960; De Grisse and Loof, 1965), *D. mineira* (Vovlas et al., 1989), *D. perseae* (Cid del Prado Vera and Loof, 1984), and *D. sinensis*. Moreover, the new species is also compared with *D. limitanea* (Luc, 1959; De Grisse and Loof, 1965), which is a widespread and common species of the genus.

It differs from *D. discolabia* by having longer body length (382–556 μm *vs.* 190–300 μm), longer stylet (59–76 μm *vs.* 35–47 μm), less number of annuli on the body (R = 80–86 *vs.* 155–174), less number of annuli between anterior end to excretory pore (Rex = 21–23 *vs.* 47–56), less number of annuli between vulva to tail terminus (RV = 9–11 *vs.* 14–17), less number of annuli between anus to tail terminus (Ran = 6–7 *vs.* 10–14), crenation on annuli absent (*vs.* present).

From *D. mauritiensis* by having longer body length (382–556 μm *vs.* 280–390 μm), longer stylet (59–76 μm *vs.* 33–38 μm), less number of annuli on the body (R = 80–86 *vs.* 140–152), less number of annuli between anterior end to excretory pore (Rex = 21–23 *vs.* 42–45), more number of annuli between vulva and anus (2–3 *vs.* 1), less number of annuli between anus to tail terminus (Ran = 6–7 *vs.* 8–10), V value (88–91 *vs.* 93–96), crenation on annuli absent (*vs.* present), absence of rudimentary submedian lobes (*vs.* present), and vulval lip morphology (anterior lip not enlarged *vs.* enlarged).

*D. limitanea* belongs to group 2 in having a labial disc with ventral and dorsal deep indentations. The new species differs from *D. limitanea* by having longer body length (382–556 μm *vs.* 160–310 μm), less number of annuli between anterior end to excretory pore (Rex = 21–23 *vs.* 30–38), anastomoses (few *vs.* numerous), and tail terminus (bilobed *vs.* bluntly rounded).

From *D. mineira* by having longer body length (382–556 μm *vs.* 250–340 μm), V value (88–91 *vs.* 93–94), VL/VB ratio (1.1–1.5 *vs.* 0.8–1.0), absence of rudimentary submedian lobes (*vs.* present), the position of excretory pore (1–2 annuli posterior to esophageal bulb *vs.* at or anterior to it), the shape of vagina (straight *vs.* curved), shape of vulval anterior lip (simple *vs.* bilobed), and tail terminus (bilobed *vs.* lobed).

From *D. perseae* by having longer body length (382–556 μm *vs.* 220–370 μm), less number of annuli on the body (R = 80–86 *vs.* 108–126), less number of annuli between anterior end to excretory pore (Rex = 21–23 *vs.* 32–38), less number of annuli between vulva to tail terminus (RV = 9–11 *vs.* 14–20), less number of annuli between anus to tail terminus (Ran = 6–7 *vs.* 9–12), the position of excretory pore (1–2 annuli posterior to esophageal bulb *vs.* at the base of it), shape of vagina (straight *vs.* sigmoid), and shape of postvulval body (elongated conoid with bilobed terminus *vs.* convex conoid, terminus subacute, and directed dorsal).

From *D. sinensis* by having more number of annuli on the body (80–86 *vs.* 64–69), absence of rudimentary submedian lobes (*vs.* present), excretory pore (conspicuous *vs.* inconspicuous), the position of excretory pore (1–2 annuli posterior to esophageal bulb *vs.* at the base of it), and tail terminus (bilobed *vs.* lobed).

### Molecular profiles and phylogenetic status

**Figure 4:**
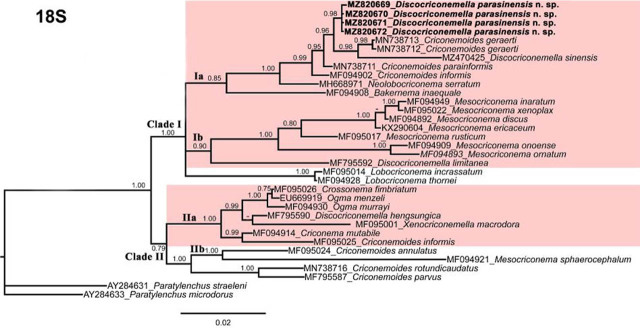
Phylogenetic relationships of *D. parasinensis* n. sp. with other criconematids species as inferred from Bayesian analysis using the 18S rRNA gene sequence dataset with the TIM1+I+G model. Posterior probability >70% is given for appropriate clades. Newly obtained sequences are indicated in bold.

**Figure 5:**
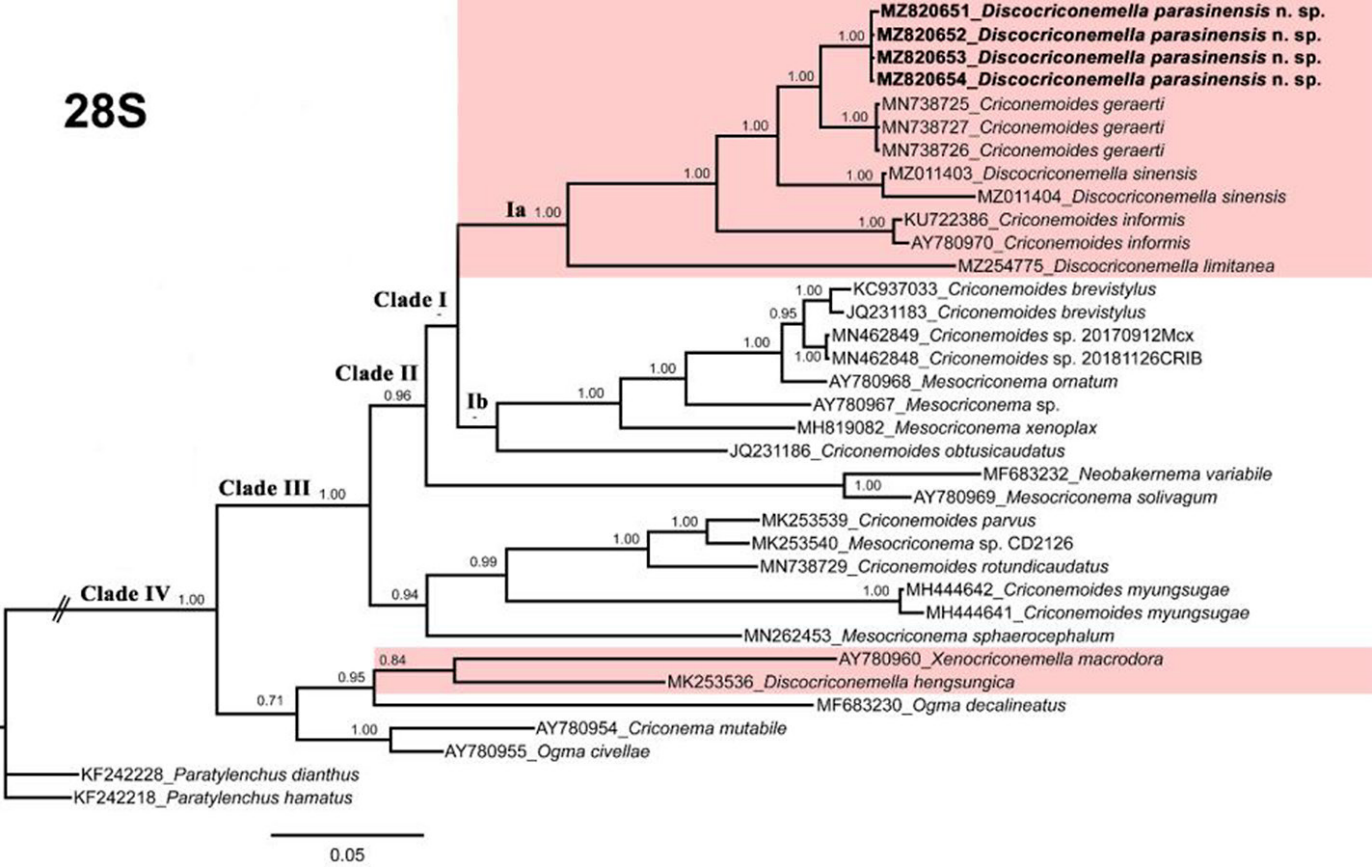
Phylogenetic relationships of *D. parasinensis* n. sp. with other criconematids species as inferred from Bayesian analysis using the D2–D3 of 28S rRNA gene sequence dataset with the TIM3+I +G model. Posterior probability >70% is given for appropriate clades. Newly obtained sequences are indicated in bold.

**Figure 6:**
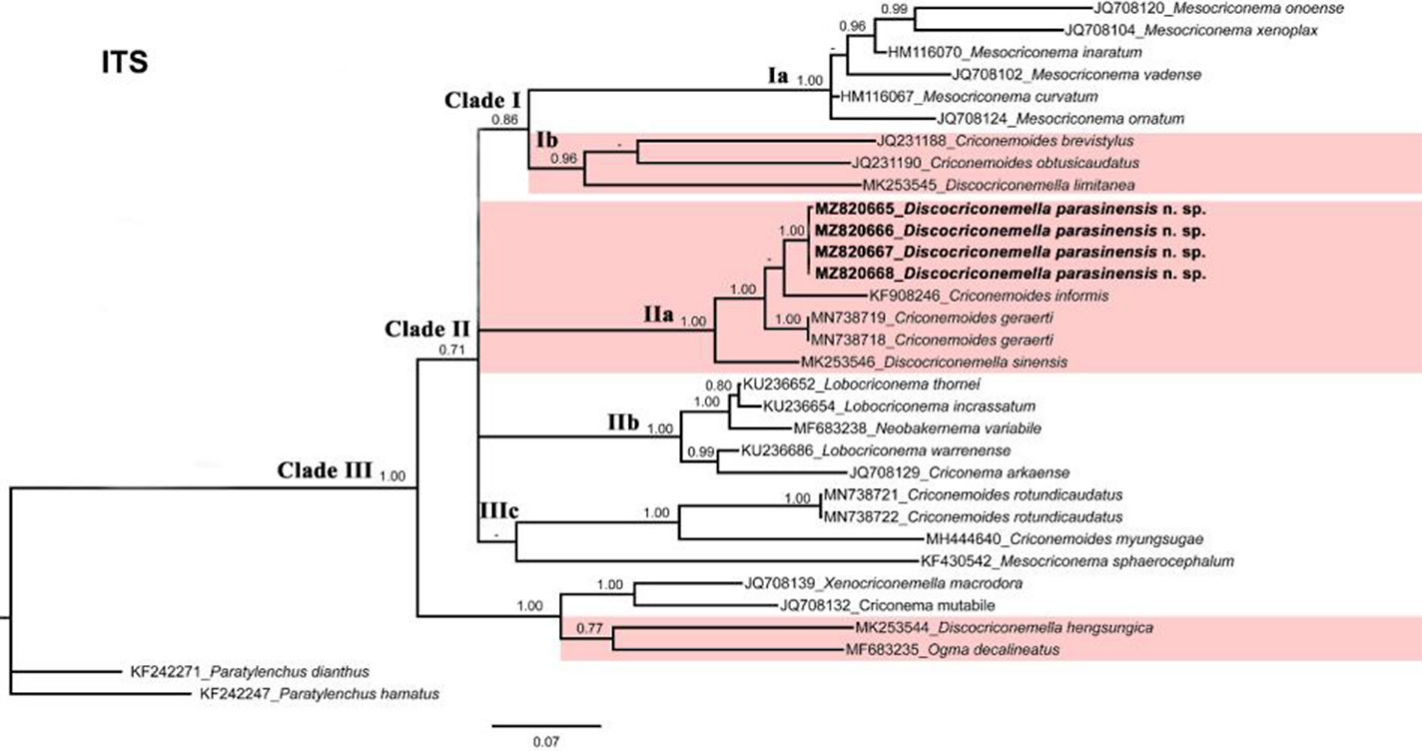
Phylogenetic relationships of *D. parasinensis* n. sp. with other criconematids species as inferred from Bayesian analysis using the ITS rRNA gene sequence dataset with the GTR+I+G model. Posterior probability >70% is given for appropriate clades. Newly obtained sequences are indicated in bold.

The new *Discocriconemella* species was molecularly characterized using 18S, D2–D3 expansion segments of 28S and ITS sequences of rRNA genes. The newly obtained sequences were deposited in the GenBank. Phylogenetic relationships among criconematid species were inferred using the aforementioned gene sequences using BI and are given in Figures 4–6, respectively.

The 18S tree (Fig. 4) was constructed from 34 criconematid taxa with *Paratylenchus straeleni* (AY284631) and *P. microdorus* (AY284633) as outgroup species. The tree has two well supported major clades. The new species is in the first clade and shows sister relationship with *Criconemoides geraerti* (MN738712-MN738713) and *D. sinensis* (MZ470425). The cosmopolitan species *D. limitanea* is in the basal position of the first clade and grouped with species of *Mesocriconema*, while *D. hengsungica* grouped with *Xenocriconemella macrodora* in the second clade. In this tree, all four *Discocriconemella* species arranged distantly from each other and showed paraphyletic relationships except *D. sinensis*.

The D2–D3 of the 28S tree (Fig. 5) was constructed from 35 criconematid taxa with *P. dianthus* (KF242228) and *P. hamatus* (KF242218) as outgroup species. The tree has four well-supported clades (PP = 0.96–1.00), the new species grouped in the first clade with *C. geraerti* (MN738725-MN738727), *C. informis* (KU722386, AY780970), *D. sinensis* (MZ011403-MZ011404), and *D. limitanea* (MZ254775) whereas *D. hengsungica* grouped with *X. macrodora* and occupied the basal clade.

The ITS tree (Fig. 6) was constructed from 32 criconematid taxa with *P. dianthus* (KF242271) and *P. hamatus* (KF242247) as outgroup species. The tree has three well supported clades, the new species grouped in the second clade with *C. geraerti* (MN738718-MN738719), *C. informis* (KF908246), and *D. sinensis* (MK253546) whereas *D. hengsungica* grouped with *Ogma decalineatus* and again occupied the basal position in the tree.

Phylogenetic analyses conducted in this study indicated that the *D. parasinensis* n. sp. closely grouped *with C. geraerti* and *C. informis* instead of its own genus members, that is, *D. hengsungica, D. limitanea*, and *D. sinensis*. In *Discocriconemella*, the presence of disc-like lip annulus is the salient character, none of the *Criconemoides* species reported to have such labial morphology. In this context, the close phylogenetic affinity of *D. parasinensis* n. sp., with *Criconemoides* species, is unresolved and at this point remains a subject to further research.

## Discussion

*Discocriconemella* species are generally reported from warmer areas of the world. The distribution of reported species of *Discocriconemella* is as follows: the majority of the species were reported from Asia (12 spp.), followed by Africa (6 spp.) and South America (6 spp.), very few species were described from Oceania (3 spp.) and North America (2 spp.) (Geraert, 2010). The host associations of *Discocriconemella* species are also not well documented, the majority of species were reported in association with grasses or woody plants (Siddiqi, 2000).

The labial arrangement of *Discocriconemella* is the main generic character distinguishing it from other genera of Family Criconematidae. However, this common morphological character of the genus does not seem to reflect in the phylogenetic positioning. In our phylogenetic analyses, *D. parasinensis* n. sp., grouped next to *Criconemoides geraerti* instead of other *Discocriconemella* species. Morphologically, both species are entirely different, their close phylogenetic affinity indicates the presence of divergent lineages in genus *Discocriconemella.* Only *D. hengsungica*, *D. limitanea*, and *D. sinensis* were described with sequence-based data, however, in all our phylogenetic analyses these species also grouped independently and distant from *D. parasinensis* n. sp. except for *D. sinensis.* These paraphyletic relationships were also observed in our previous studies (Maria et al., 2018a, 2019), however, due to the scarcity of *Discocriconemella* sequences such phylogenetic positioning for *D. parasinensis* n. sp. and other members cannot be completely clarified. A similar taxonomic puzzle was observed by Qiao et al. (2018) while describing a new species of genus *Labrys*—the authors reported that *L. fujianensis* is sister to *Miculenchus* and is never closely related to other *Labrys* species. Disconcordance of 18S and 28S rDNA phylogenies have been observed by Qing et al. (2018) while studying the phylogeny of Tylenchidae taxa and reported that the phylogenetic position of *Miculenchus* is not straightforward for example in 28S rRNA phylogeny, it has a sister relationship to *Malenchus* whereas, in the 18S rRNA phylogeny, *Miculenchus* is sister to *Lelenchus* and thus divergent from *Malenchus*. Consequently, divergent positioning of species of the same genus is not unexpected and can be elucidated in detail, once the molecular information of all the member species becomes available.

Since our phylogenetic analyses are not very conclusive, based on species’ morphology, we recognize *D*. *parasinensis* n. sp. in group 1 of *Discocriconemella* species. The *Discocriconemella* grouping was proposed by Vovlas (1992) and divided the genus into four different groups based on the labial disc morphology. The brief definition of each group is as follows: labial disc round with uninterrupted margins (group 1); disc with ventral and dorsal deep indentations (group 2); disc indentations giving a four-lobed appearance (group 3); and rounded disc with paired dorsal and ventral projections (group 4). The *Discocriconemella* species included in our phylogenetic analyses comes in three different groups (such as *D. parasinensis* n. sp. and *D. sinensis* – group 1, *D. limitanea* – group 2, *D. hengsungica* – group 4) and hold a unique phylogenetic position in our trees. Therefore, we agree with other nematologists that morphological grouping was only established for the convenience of identification and does not always reflect the evolutionary history (Gutiérrez-Gutiérrez et al., 2013; Tzortzakakis et al., 2014; Handoo et al., 2016; Maria et al., 2018b).

Despite the frequent abundance of criconematids in the rhizosphere of natural vegetation, their life histories and feeding habits are not well studied (Maria et al., 2018a, 2019). This could be due to the reason that the majority of criconematids were reported from the grasslands, they have never been associated with agricultural production areas. Based on criconematids robust and long stylets they are either considered as epidermal or cortical feeders of higher plants (Siddiqi, 2000). In the present study, *D. parasinensis* n. sp. was recovered in lower density which suggests that as of yet, it is a mild parasitic species and do not behave as potential pest. In addition to that, the presence of *D. parasinensis* n. sp. in the rhizosphere of Tea plants (*Camellia sinensis*) indicated that the known diversity of *Discocriconemella* has increased and this type of vegetation harboring much more nematofauna that has been expected to be discovered.
